# Antiphospholipid Syndrome in Chronic Thromboembolic Pulmonary Hypertension and Chronic Thromboembolic Pulmonary Disease without Pulmonary Hypertension: Prevalence and Clinical Impact in a Dutch Cohort

**DOI:** 10.1055/a-2938-6324

**Published:** 2026-09-04

**Authors:** Tamara C. Rodenburg, Linde J. op de Hoek, Coen Van Kan, Jurjan Aman, Esther Nossent, Anton Vonk Noordegraaf, Harm Jan Bogaard, Frederikus A. Klok, Lilian J. Meijboom, Josien Van Es, Thijs E. van Mens

**Affiliations:** 1 Department of Cardiovascular Sciences, Pulmonary Hypertension and ThrombosisAmsterdam UMC Location Vrije UniversiteitAmsterdam110Netherlands; 2 Department of Medicine-Thrombosis and Hemostasis 4501 Leiden University Medical CenterLeidenSouth HollandNetherlands; 3 Department of Respiratory Medicine 10215 OLVGAmsterdamNetherlands; 4 Department of Radiology and Nuclear MedicineAmsterdam UMC Location Vrije UniversiteitAmsterdamNoord-HollandNetherlands

**Keywords:** thrombosis, antiphospholipid antibodies, pulmonary endarterectomy, balloon pulmonary angioplasty

## Abstract

Antiphospholipid syndrome (APS) is a prothrombotic autoimmune disorder associated with venous thromboembolism (VTE) and chronic thromboembolic pulmonary hypertension (CTEPH). Current guidelines recommend APS screening in CTEPH patients. The exact prevalence of APS in both CTEPH and chronic thromboembolic pulmonary disease (CTEPD) without pulmonary hypertension (PH) remains unclear. Although recently increasing attention has been directed toward treatment outcomes following pulmonary endarterectomy (PEA), other CTEPD without PH patients and those treated with alternative interventions, such as balloon pulmonary angioplasty or PH medication, remain insufficiently studied. This study aimed to assess the prevalence, lesion distribution, and outcomes of APS in CTEPH and CTEPD without PH. We retrospectively analyzed 259 patients diagnosed with CTEPH ( *n* = 233) or CTEPD without PH ( *n* = 26) at a tertiary care referral center in the Netherlands. Demographic, clinical, hemodynamic, laboratory, and follow-up data were collected from medical records. Lesion distribution in APS patients was related to literature. In 140 out of 259 patients, APS testing results could be retrieved. Among these, 112/140 (80%) had no APS, 19/140 (14%) tested positive without repeated confirmation or without APS clinical criteria, and 9/140 (6%) were classified as APS. Of the nine APS patients, four had a triple antibody profile. APS patients were relatively young (mean age 44 years), often had autoimmune comorbidities (3/9; 33%) and a history of recurrent VTE (6/9; 67%). All APS patients experienced mild thrombocytopenia post-PEA. Both proximal (5/9; 56%) and distal disease (6/9; 67%) were observed in APS. This study provides a real-world overview of CTEPH/CTEPD without PH patients with and without APS and contributes to the limited literature on this specific patient population.

## Introduction

Chronic thromboembolic pulmonary hypertension (CTEPH) is a progressive disease of the pulmonary vasculature, characterized by persistent organized thrombi and secondary vascular remodeling, leading to elevated pulmonary vascular resistance (PVR), right ventricular failure, and premature mortality. 1 Treatments include pulmonary endarterectomy (PEA), balloon pulmonary angioplasty (BPA), and PH-targeted medication. Over the last decade, recognition of patients with chronic thromboembolic pulmonary disease (CTEPD) without PH has accelerated. 2 3

Despite advances in diagnosis and treatment, the pathogenesis of CTEPH remains incompletely understood. Although traditionally regarded as a complication of acute pulmonary embolism (PE), many patients lack a documented history of venous thromboembolism (VTE), and radiological signs of CTEPH frequently present on computed tomography pulmonary angiography (CTPA) at the time of the initial PE diagnosis, suggesting other mechanisms beyond unresolved PE. 4 5 6 7 8 9 10 Several potential risk factors associated with CTEPH have been identified, 11 including antiphospholipid syndrome (APS). APS is an autoimmune prothrombotic disorder characterized by thrombosis and/or pregnancy morbidity, in the presence of persistent antiphospholipid antibodies (aPL), and lupus anticoagulant (LAC), anticardiolipin (aCL), and anti-β2-glycoprotein I (aβ2GPI). 12 13 While APS is a well-established risk factor for VTE and is associated with CTEPH, hereditary thrombophilias (e.g., antithrombin, protein C/S deficiency, and factor V Leiden) are not. 14

Remarkably, CTEPH itself is not classified as a thrombotic APS manifestation under either the 2006 or 2023 APS classification criteria. 12 13 Current PH guidelines recommend routine aPL screening in CTEPH patients, and recommend the use of vitamin K antagonists (VKAs) over direct oral anticoagulants (DOACs) in APS. 15 This seems to be regardless of the presence of a previous thrombotic event. In contrast, acute PE guidelines suggest testing only in patients who present with VTE at a young age, without identifiable risk factors, and especially in case of a strong family history of VTE. 16 Besides the guideline-recommended switch to VKAs, the downstream clinical consequences of identifying APS in the CTEPH population remain poorly defined.

Reported aPL positivity in CTEPH varies widely, 5 17 18 19 20 21 22 23 24 25 with a meta-analysis estimating a pooled prevalence of 11.8%. 26 However, most included studies relied on single aPL measurements, which may include transient antibody positivity. Transient aPL may occur in the context of infections and resolve without clinical manifestations, which is why APS criteria require aPL positivity confirmation at least 12 weeks apart, and the presence of specific clinical manifestations. Until recently, the study by Jiang et al. was the only study that reported APS prevalence using repeated aPL measurements in CTEPH, demonstrating a repeated aPL positivity rate of 7.7%, although without applying formal APS classification criteria. 25 Given geographical differences in the epidemiology of VTE, CTEPH, and APS, 27 28 29 the applicability of these findings to European populations remains uncertain. More recently, two studies were published in which formal APS classification criteria were applied, and postoperative PEA complications were comprehensively evaluated. 30 31 In current practice, perioperative PEA protocols for APS patients often differ from those for no APS patients, as pro-inflammatory activation of aPL may contribute to hypercoagulability during cardiopulmonary bypass and potentially reduce the response to heparin. 32

The aims of this study were to provide real-world data from an unselected, consecutive cohort to: (1) determine the prevalence of APS in patients with CTEPH and CTEPD without PH, including APS profiles; (2) assess clinical characteristics, treatment allocation, outcomes of both PEA and BPA, and complications in patients with and without APS; and (3) evaluate thrombus localization in APS-associated disease in relation to current literature.

## Methods

### Study Design and Population

This retrospective observational study included all consecutive patients diagnosed with CTEPH and CTEPD without PH ( *n* = 259) between January 2017 and December 2024 identified from the PH registry at Amsterdam UMC, a tertiary referral center for CTEPH in the Netherlands. Standardized diagnostic workup included right heart catheterization (RHC), CTPA, and measurement of NT-proBNP. For the first study objective, aPL measurements were extracted from the clinical records of the included patients, when available. For the second objective, clinical characteristics, RHC parameters, World Health Organization functional class (WHO FC), laboratory results (e.g., NTproBNP and aPL), and APS classifying events were extracted from clinical records. Peri- and postprocedural complications of PEA and BPA were recorded, along with follow-up data, including WHO FC, NT-proBNP, and RHC parameters, at 6 months post-intervention. As this study was conducted using data obtained during routine clinical care, it did not fall within the scope of the Medical Research Involving Human Subjects Act and informed consent was not required.

### APS Diagnosis

For the first objective of this study, APS was classified according to both the 2006 and 2023 international criteria requiring persistent aPL positivity (≥12 weeks apart) in combination with a qualifying clinical manifestation. 12 13 The aCL and aβ2GPI antibodies (IgG and/or IgM) were measured using EliA assays (Phadia 250, Thermo Fisher Scientific), applying a cut-off value of 10 units for aβ2GPI and 33 units for aCL, according to the 99th percentile of a healthy control group. LAC was detected according to International Society on Thrombosis and Haemostasis guidelines. 33 For patients using a DOAC, an activated charcoal-based DOAC removal method (DOAC-Remove, 5-Diagnostics AG) was used prior to LAC testing. A triple-positive antibody profile was defined as LAC + , aCL + , and aβ2GPI +. Patients were categorized into four groups: (1) APS, meeting both laboratory and clinical criteria; (2) no APS; (3) possible APS, aPL positivity at a single time point without repeated confirmation, or without APS clinical criteria; and (4) no APS results available.

### Diagnosis and Treatment

Diagnosis and treatment allocation were determined by a multidisciplinary team including PH expert pulmonologists and radiologists, a thoracic surgeon, and a vascular medicine specialist. Patients with organized thromboembolic lesions without PH on RHC were classified as CTEPD without PH. Treatments included PEA or BPA, with or without PH medication; PH medication alone; or no treatment. Patients receiving only PH medication or no PH treatment, as well as those who underwent both PEA and BPA, were excluded from the analyses of treatment outcomes and complications of the second study objective. Procedural details for PEA and BPA have been published previously. 34 35 36

Perioperative anticoagulation management followed institutional protocols. For PEA, oral anticoagulation was discontinued 7 days preoperatively and bridged with therapeutic low-molecular-weight heparin (LMWH). In patients without APS, LMWH was withheld on the day before surgery, whereas in patients with APS, it was continued until the morning of admission. Postoperatively, unfractionated heparin was initiated, aiming for an activated partial thromboplastin time (aPTT) of 45–60 s in patients without APS, and 50–70 s in patients with APS.

For BPA, peri-procedural anticoagulation management did not differ between patients with and without APS. DOACs were withheld prior to the procedure according to renal function, while VKAs were discontinued 5 days before BPA and bridged with LMWH until the day before the procedure. Oral anticoagulation (VKA or DOAC) was restarted on the evening of the procedure.

### Right Heart Catheterization

Right heart catheterization (RHC) was performed according to standard protocols. 37 38 Recorded parameters included mean pulmonary arterial pressure (mPAP), pulmonary arterial wedge pressure (PAWP), mean right atrial pressure (mRAP), cardiac index (CI; cardiac output with thermodilution indexed to body surface area), arterial oxygen saturation (SaO _2_ ), mixed venous oxygen saturation (SvO _2_ ), and PVR (calculated as 80 × [mPAP − PAWP]/CO, dyn·s·cm ^−5^ ). In patients with PAWP > 15 mm Hg, left ventricular end-diastolic pressure was assessed to distinguish pre- from post-capillary PH.

### Imaging Analysis of Thromboembolic Lesions

For the third study objective, pre-procedural CTPA scans of APS patients were retrospectively reviewed by an experienced CTEPH radiologist. Lesion distribution was assessed using the classification approach described by Jiang et al., based on the University of California, San Diego surgical classification. 25 39 Thromboembolic lesions in the left and right pulmonary arteries were categorized according to the most proximal level of obstruction, with levels I to II defined as proximal and levels III to IV as distal disease. Imaging analysis was limited to patients with APS, as comprehensive CTPA reassessment of the entire cohort was not feasible given available staffing.

### Statistical Analysis

This study was descriptive in nature; no inferential statistical analyses were performed. Continuous variables were presented as mean ± standard deviation (SD) or median (interquartile range [IQR]; 25th–75th percentile) depending on distribution. Categorical variables are presented as counts (%). Normality was assessed via histograms. For the imaging analysis, data were described with counts (%) and related to previous literature. Missing data were not imputed. Analyses were performed in R (version 4.5.1).

## Results

### Study Population

**Fig. 1:**
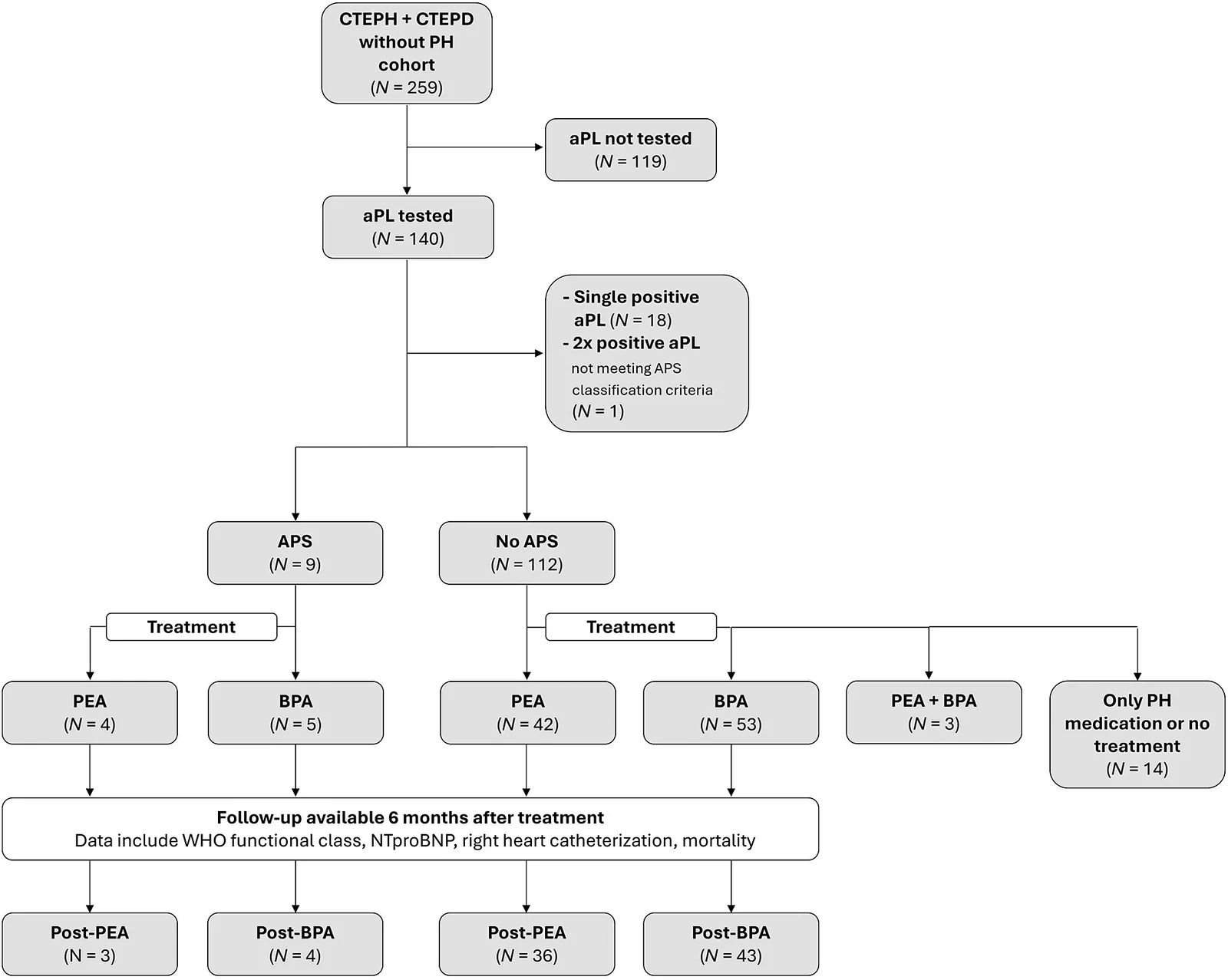
Flowchart with the number of included patients, treatment, and follow-up. aPL, antiphospholipid antibodies; APS, antiphospholipid syndrome; BPA, balloon pulmonary angioplasty; CTEPD, chronic thromboembolic pulmonary disease; CTEPH, chronic thromboembolic pulmonary hypertension; PEA, pulmonary endarterectomy; PH, pulmonary hypertension; WHO, World Health Organization.

A total of 259 patients were included, of whom 233 (90%) had CTEPH and 26 (10%) had CTEPD without PH. aPL test results were available in 140 out of 259 patients (54%). Among these 112 (80%) had no APS, 19 (14%) had possible APS, and nine (6%) had APS ( Fig. 1 ). In five out of the nine APS patients (56%), APS had already been established before the CTEPH diagnosis. In the other four APS patients (44%), APS was established during or after CTEPH diagnosis.

### Baseline Characteristics

Baseline demographics and comorbidities are summarized in Table 1 . The median age at CTEPH/CTEPD without PH diagnosis was 44 years (34–74) in patients with APS, and 64 years (16–84) in patients without APS. VKAs were used by 21 out of 112 patients (19%) in the no APS group, and seven out of nine patients (78%) in the APS group, of which three out of seven (43%) were switched to a VKA during the CTEPH workup because of their APS status. A history of autoimmune disease other than APS was documented in three out of nine (33%) APS patients and in seven out of 112 (6%) no APS patients. Recurrent VTE was reported in six out of nine (67%) APS patients and in 38 out of 112 (34%) no APS patients. DVT occurred in seven out of nine (78%) APS patients and 31 out of 112 (28%) no APS patients.

**Table 1:** Baseline characteristics.

	APS ( *N* = 9)	No APS ( *N* = 112)
Age in years at diagnosis, median (IQR)	44 (34–74)	64 (16–84)
Female	5 (56)	43 (38)
BMI, median (IQR)	28 (26–30)	27 (24–31)
Anticoagulation		
DOAC	2 (22)	91 (81)
VKA	7 (78)	21 (19)
Smoker		
Never	4 (44)	47 (42)
Current + Former	5 (56)	61 (55)
Unknown	0	4 (4)
Blood group, non-O	5 (56)	37 (33)
Malignancy	0	19 (17)
Pulmonary comorbidities ^a^	2 (22)	40 (36)
Other autoimmune disorder	3 (33)	7 (6)
Diabetes	1 (11)	12 (11)
Splenectomy	0	6 (5)
Thyroid hormone suppletion	0	7 (6)
Systemic hypertension	2 (22)	35 (31)
Cardiac comorbidities		
Atrial fibrillation	0	5 (5)
Left-sided heart failure	0	1 (1)
Ischemic heart disease	0	3 (3)
Other	0	10 (9)
TIA or iCVA	1 (11)	9 (8)
History of reported acute VTE		
PE	7 (78)	99 (88)
DVT	7 (78)	31 (28)
Recurrent VTE	6 (67)	38 (34)
No history of VTE	0	12 (11)

Abbreviations: APS, antiphospholipid syndrome; BMI, body mass index; DOAC, direct oral anticoagulant; DVT, deep venous thrombosis; iCVA, ischemic cerebrovascular accident; IQR, interquartile range; PE, pulmonary embolism; TIA, transient ischemic attack; VKA, vitamin K antagonist; VTE, venous thromboembolism.

Notes: Data are presented as *N* (%), unless stated otherwise.

^a^ Chronic obstructive pulmonary disease (COPD), asthma, obstructive sleep apnea (OSA), interstitial lung disease.

### APS Profiles

The clinical and serological characteristics of the nine APS patients are detailed in Table 2 . LAC was present in all patients. A triple-positive antibody profile was observed in four patients (44%), while three (33%) demonstrated double antibody positivity and two (22%) had single antibody positivity. All patients had a history of VTE, and two (22%) additionally had arterial or obstetric manifestations. Eight patients fulfilled both the 2006 and 2023 APS classification criteria, whereas one patient fulfilled the 2006 criteria only. 12 13

**Table 2:** Antiphospholipid syndrome specification.

	LAC	Antibody profile	Previous APS manifestations	APS classification criteria (2006 and/or 2023)	CTEPH or CTEPD without PH
Patient 1	Positive	Single	Venous	2023 and 2006	CTEPH
Patient 2	Positive	Double	Venous	2023 and 2006	CTEPH
Patient 3	Positive	Triple	Venous, obstetric	2023 and 2006	CTEPH
Patient 4	Positive	Double	Venous	2023 and 2006	CTEPH
Patient 5	Positive	Single	Venous	2023 and 2006	CTEPH
Patient 6	Positive	Triple	Venous, arterial, obstetric	2023 and 2006	CTEPD without PH
Patient 7	Positive	Triple	Venous	2006 ^a^	CTEPD without PH
Patient 8	Positive	Double	Venous	2023 and 2006	CTEPH
Patient 9	Positive	Triple	Venous	2023 and 2006	CTEPH

Abbreviations: APS, antiphospholipid syndrome; CTEPD, chronic thromboembolic pulmonary disease; CTEPH, chronic thromboembolic pulmonary hypertension; LAC, lupus anticoagulant; PH, pulmonary hypertension.

Notes: The patient who tested positive for aPL twice, but did not meet any classification criteria, had a single antibody profile, was LAC negative, and had CTEPH.

^a^ This patient had one thrombotic event with a high-risk VTE profile, characterized by multiple VTE risk factors present at the time of the thrombotic event, and therefore did not meet the 2023 APS classification criteria.

### CTEPH Characteristics, Treatment Allocation, and Treatment Response

Hemodynamic and functional data are presented in Table 3 . Among nine patients classified as APS, four (44%) underwent PEA and five (56%) underwent BPA. In the no APS group ( *n* = 122), 42 (38%) were treated with PEA and 53 (47%) with BPA ( Fig. 1 ). In addition, 14 (13%) received no interventional treatment or medical therapy only, and three (3%) underwent both PEA and BPA.

**Table 3:** Hemodynamic and functional parameters at baseline (diagnosis) and follow-up (6 months after treatment), stratified according to treatment (pulmonary endarterectomy or balloon pulmonary angioplasty).

PEA				
	APS—baseline ( *N* = 4)	No APS—baseline ( *N* = 42)	APS—follow-up ( *N* = 3)	No APS—follow-up ^a^ ( *N* = 36)
WHO FC ^b^				
I	0	0	1 (33)	7 (19)
II	0	17 (41)	2 (67)	26 (72)
III	4 (100)	23 (55)	0	3 (8)
IV	0	2 (5)	0	0
NTproBNP ^c^ (ng/L)	690 (178–2,073)	346 (100–1,225)	158 (141–278)	174 (126–423)
RHC				
mRAP (mmHg) ^c^	10 (8–14)	8 (6–10)	4 (4–4)	6 (4–8)
mPAP (mmHg) ^c^	44 (3)	43 (13)	27 (11)	26 (11)
PAWP (mmHg) ^d^	10 (6)	12 (4)	10 (5)	12 (4)
CI (L/min/m ^2^ ) ^d^	2.1 (0.8)	2.5 (0.6)	3.7 (0.4)	3.0 (0.7)
PVR (dyn·s·cm ^−5^ ) ^c^	596 (482–799)	441 (250–748)	184 (142–210)	130 (107–198)
SaO _2_ (%) ^d^	94 (2)	92 (5)	95 (3)	97 (2)
SvO _2_ (%) ^d^	59 (7)	63 (9)	71 (5)	68 (6)
CTEPD without PH	0	2 (5)	0	2 (6)
Mortality ^b^	–	–	0	1 (3)

BPA				
	APS—baseline ( *N* = 5)	No APS—baseline ( *N* = 53)	APS—follow-up ^e^ ( *N* = 4)	No APS—follow-up ^f^ ( *N* = 43)
WHO FC ^b^				
I	0	0	0	7 (16)
II	1 (20)	11 (21)	3 (75)	26 (60)
III	4 (80)	41 (77)	1 (25)	10 (23)
IV	0	1 (2)	0	0
NTproBNP ^c^ (ng/L)	193 (142–291)	622 (92–1,805)	109 (65–210)	152 (58–776)
RHC				
mRAP (mmHg) ^c^	6 (6–6)	6 (5–9)	7 (7–7)	6 (5–8)
mPAP (mmHg) ^c^	39 (21)	43 (14)	29 (7)	33 (11)
PAWP (mmHg) ^d^	11 (2)	11 (3)	13 (2)	12 (3)
CI (L/min/m ^2^ ) ^d^	3.4 (1.1)	2.6 (0.7)	2.9 (0.5)	3.2 (0.7)
PVR (dyn·s·cm ^−5^ ) ^c^	204 (133–291)	455 (308–724)	218 (186–233)	231 (175–327)
SaO _2_ (%) ^d^	95 (5)	92 (4)	96 (3)	92 (5)
SvO _2_ (%) ^d^	72 (15)	64 (8)	73 (5)	65 (9)
CTEPD without PH	2 (40)	5 (9)	1 (25)	3 (7)
Mortality ^b^	–	–	0	0

Abbreviations: APS, antiphospholipid syndrome; BPA, balloon pulmonary angioplasty; CI, cardiac index; CTEPD, chronic thromboembolic pulmonary disease; mPAP, mean pulmonary artery pressure; mRAP, mean right atrial pressure; PAWP, pulmonary artery wedge pressure; PEA, pulmonary endarterectomy; PH, pulmonary hypertension; PVR, pulmonary vascular resistance; RHC, right heart catheterization; SaO _2_ , arterial oxygen saturation; SvO _2_ , venous oxygen saturation; WHO FC, World Health Organization Functional Class.

Notes: ^a^ NTproBNP was available for 35 patients; RHC was available for 27 patients.

^b^*N* (%).

^c^ Median (interquartile range).

^d^ Mean (standard deviation).

^e^ NTproBNP was available for four patients; RHC was available for three patients.

^f^ NTproBNP was available for 32 patients; RHC was available for 24 patients.

At baseline, most patients were in WHO FC III, including eight out of nine (89%) APS patients and 65 out of 95 (68%) patients without APS. In patients with APS, baseline PVR was 382 dyn·s·cm ^−5^ (204–676), while patients without APS showed PVR levels of 468 dyn·s·cm ^−5^ (259–757) ( **Supplementary Table S3** (online only)). CTEPD without PH was identified in two out of nine (22%) APS patients, both of whom were treated with BPA. Eleven out of 112 (10%) of the no APS patients were CTEPD without PH patients, of whom five were managed with BPA (45%), two with PEA (18%), and four (36%) did not receive PH therapy. Baseline data of only CTEPH patients are summarized in **Supplementary Table S3** (online only). At six months follow-up, a trend toward improved hemodynamics was observed in both groups, with six out of seven (86%) APS patients and 66 out of 79 (84%) no APS patients classified as WHO FC I–II. No mortality was observed among APS patients and one death occurred in the no APS group post-PEA.

### Patients Without APS Testing or Possible APS

Patients without APS test results ( *n* = 119) and patients with possible APS ( *n* = 19) with positive aPL once without repeated confirmation ( *n* = 18) are summarized in **Supplementary Tables S1 and S2** (online only). Within the possible APS group, single aPL positivity was observed in 16 patients, while double ( *n* = 2) and triple ( *n* = 1) positivity were observed less frequently ( **Supplementary Table S4** (online only)).

Of the possible APS group, one patient, with a history of systemic hypertension and pulmonary comorbidities, demonstrated persistent single ab2GPI positivity in the absence of documented thrombotic events other than CTEPH, and could therefore not officially be classified as having APS. An LAC determination was not performed due to use of a DOAC at the time of testing. Hemodynamic assessment demonstrated severe PH with an mPAP of 60 mm Hg and PVR of 1538 dyn·s·cm ^−5^ . The patient was classified as WHO functional class III.

### Peri-interventional Complications

Following PEA, peri-interventional complications were documented in all APS patients and 34 out of 42 (81%) no APS patients. In the APS group, severe thrombocytopenia (<50 × 10 ^9^ /L) occurred in one out of four patients (25%), due to heparin-induced thrombocytopenia (HIT). Mild thrombocytopenia (50–100 × 10 ^9^ /L) was observed in three out of four (75%) APS patients and in 14 out of 42 (33%) patients without APS. No cases of stroke or acute respiratory distress syndrome were reported in either group ( Table 4 ).

**Table 4:** Chronic thromboembolic pulmonary hypertension treatment and complications.

PEA		
	APS ( *N* = 4)	No APS ( *N* = 42)
Complications ^a^	4 (100)	34 (81)
Infection	0	7 ^b^ (17)
Cardiac arrhythmia ^c^	0	17 (41)
Pulmonary hemorrhage	0	2 (5)
Thrombocytopenia (50–100 × 10 ^9^ /L)	3 (75)	14 (33)
Thrombocytopenia (<50 × 10 ^9^ /L)	1 (25)	2 (5)
HIT	1 (25)	0
VA-ECMO	0	2 (5)
Cardiac tamponade	0	5 (12)
Pericardial effusion	0	2 (5)
Pneumothorax	0	2 (5)
Neuropsychiatric complications	0	6 (14)
Acute pulmonary embolism	0	1 (2)
Other	1 ^d^ (25)	8 ^e^ (19)
Total length of stay hospital (days) ^f^	11 (9–14)	12 (9–16)
Length of stay ICU (days) ^f^	3 (2–5)	4 (2–6)

BPA		
	APS ( *N* = 5)	No APS ( *N* = 53)
Number of BPA ^f^	6 (5–6)	5 (4–6)
Complications	2 (40)	27 (51)
Hemoptysis	2 (40)	18 (34)
Vascular injury	0	6 (11)
Cardiac arrhythmia ^c^	0	2 (4)
Access site	0	5 (9)
Pneumothorax	0	1 (2)
Acute pulmonary embolism	0	1 (2)
Other ^g^	0	2 (4)

Abbreviations: APS, antiphospholipid syndrome; ARDS, acute respiratory distress syndrome; BPA, balloon pulmonary angioplasty; HIT, heparin-induced thrombocytopenia; ICU, intensive care unit; PEA, pulmonary endarterectomy; PH, pulmonary hypertension; VA-ECMO, venous-arterial extracorporeal membrane oxygenation.

Notes: Data are presented as *N* (%), unless stated otherwise.

^a^ No patients developed ARDS or stroke as postoperative complications.

^b^ Five hospital acquired pneumonia, two other.

^c^ Atrial fibrillation in 16 out of 17 PEA patients and 1 out of 2 BPA patients.

^d^ Subarachnoid hemorrhage.

^e^ Two times systemic inflammatory response syndrome (SIRS) without infection, hypokalemia, obstructive shock due to right ventricle failure, elevated liver enzymes, vocal cord paresis, acute on chronic kidney insufficiency, reperfusion edema.

^f^ Median (interquartile range).

^g^ Increase in NTproBNP and air embolus.

Following BPA, complications were reported in two out of five (40%) APS patients and in 27 out of 53 (51%) patients without APS. No cases of catastrophic APS were observed following PEA or BPA ( Table 4 ).

### Distribution of Thromboembolic Disease Localization in APS Patients

Among the nine APS patients, all demonstrated bilateral thromboembolic involvement. Two (22%) APS patients showed exclusively proximal (levels I–II) disease, while four (44%) showed exclusively distal disease (levels II–IV). Three (33%) showed a combination of both proximal and distal disease in the left and right pulmonary artery.

## Discussion

In this retrospective analysis of patients with CTEPH or CTEPD without PH our main findings were: (1) the prevalence of confirmed APS was 6%, with 44% of APS patients demonstrating triple aPL positivity, all including LAC positivity; (2) APS patients were relatively young with a frequent history of recurrent VTE, and commonly developed mild perioperative thrombocytopenia post-PEA; and (3) both proximal and distal chronic thromboembolic lesions were observed at CTPA in APS patients.

The prevalence of confirmed APS in our cohort (6%) was in between prevalences reported by Miard et al. (4%) and Jiang et al. (8%). 25 30 Our estimate does not include patients classified as possible APS, among whom additional APS cases are likely. The reported studies do not provide insights into patients with uncertain APS status, after testing positive once for aPL, which in clinical practice is a scenario that does occur with relevant frequency, as our results demonstrate. Within the confirmed APS group, 44% demonstrated triple aPL positivity, which is higher than that reported in a large VTE cohort (16%), 40 but lower than the 75% reported in Jiang et al.’s Chinese cohort. 25 Most patients who were tested for aPL once in our cohort had single aPL positivity, with only one case of triple positivity, suggesting a potential selection bias toward testing confirmation in patients with multiple antibody positivity. Furthermore, randomized controlled trials comparing DOACs and VKAs in APS patients demonstrated inferior outcomes with DOACs in high-risk, triple-positive APS, whereas evidence for patients with single antibody positivity remains limited. 41 42 43 This may have reduced clinicians’ incentive to repeat aPL testing in patients with single-antibody positivity, thereby contributing to the low rate of confirmatory testing observed in clinical practice.

APS patients in our cohort were relatively young and frequently presented with other autoimmune comorbidities and recurrent VTE, consistent with previous literature. 25 30 44 This high incidence of recurrent VTE may reflect a more severe APS phenotype and could have contributed to the early onset of CTEPH observed in this group. Notably, all APS patients were LAC-positive, an antibody profile known to be strongly associated with recurrent thrombosis. 45 A more favorable hemodynamic profile in APS CTEPH patients at baseline has been previously reported, 25 30 a trend also observed in our BPA-treated patients, although the sample size was small. Hemodynamic improvement after PEA in APS CTEPH patients has been reported previously, 30 31 46 47 48 and a similar trend was observed in our cohort for both PEA and BPA, although again interpretation of this trend is limited by the small sample size.

When evaluating complications rates after PEA, mild thrombocytopenia was frequently observed among APS patients, consistent with prior studies. 30 31 46 47 One APS patient in our cohort with thrombocytopenia was also diagnosed with HIT, a finding of interest in light of a recent report of overlapping auto-antigen specificity. 49 Thrombocytopenia in APS, often of autoimmune origin, is typically mild to moderate, occurs in 20 to 50% of patients, and is rarely associated with bleeding. 46 Miard et al., who investigated APS in 1,354 PEA patients at an intensive care unit (ICU), found severe thrombocytopenia to be the only complication associated with high-titer APS in CTEPH. 30 Although baseline characteristics were comparable, in our cohort neurological complications were not more frequently observed in APS patients, contrary to their findings. While Miard et al. demonstrated comparable early outcomes following PEA, their analysis was limited to ICU follow-up (median 7 days). Subsequent data from a large UK cohort extended these findings by showing that long-term rethrombosis rates and survival up to 5 years were comparable between post-PEA CTEPH patients with and without APS, although their definition of APS remains unclear. 50 Collectively, these findings support that PEA can be performed safely in patients with APS.

Regarding thromboembolic disease distribution, our findings differ from those reported by Jiang et al., who observed predominantly proximal disease (levels I–II) in 84% of APS patients. 25 In our cohort, proximal disease was present in 56% of APS patients, with a substantial proportion demonstrating exclusively distal or mixed disease patterns. In addition, Jiang et al. reported unilateral disease in 13% of CTEPH patients with APS, whereas all APS patients in our cohort exhibited bilateral involvement.

Persistent aPL positivity alone is insufficient for an APS diagnosis in the absence of a clinical manifestation. In our cohort, one patient repeatedly tested positive for aPL but had no documented history of thrombotic events other than CTEPH. Notably, other studies also showed that not all CTEPH patients with persistent aPL had a documented history of VTE. 30 31 This case highlights an important unanswered question whether to regard CTEPH or CTEPD without PH as an APS classifying event. Despite the plausible pathophysiological relation, CTEPH was not considered by the expert panel when compiling ACR/EULAR endorsed classification criteria. 12 Although the underlying mechanism remains incompletely understood, aPL increases thrombotic potential, leading to (recurrent) VTE and therefore possibly CTEPH. 51 However, 25% of CTEPH patients present without a documented history of acute VTE. 5 7 While undiagnosed or asymptomatic events may account for some cases, alternative mechanisms, such as in situ thrombosis, possibly triggered by aPL-induced chronic inflammation, 52 53 are increasingly recognized in CTEPH pathogenesis. 8 51 54 Several inflammatory and prothrombotic pathways in APS also overlap with mechanisms described in CTEPH, including activation of monocytes and neutrophils, and reduced endothelial nitric oxide synthase activity, which may be directly inhibited by aPL antibodies. 53 55 56 57 Consistently, studies in patients with Systemic Lupus Erythematosus showed an association between aPL positivity and PH. 58 In pulmonary arterial hypertension specifically, this association was present irrespective of prior thrombosis, suggesting a potential role for aPL in contributing to PH through mechanisms beyond thromboembolism, such as endothelial proliferation and vascular remodeling. This raises the question of whether aPL may be directly involved in the development of PH and CTEPH specifically.

Although we investigated this specific group of patients, applying both latest PH guidelines and the 2006 and the recently revised 2023 APS classification criteria, whereas most previous studies relied solely on single aPL positivity, 5 17 18 19 20 21 22 23 our study merits some consideration. Due to the retrospective and observational design of this study, included data were limited to what was reported in clinical records. Moreover, this study includes a relatively small number of confirmed APS patients. Although small sample sizes are inherent to studying a rare condition, aPL testing was performed less frequently than anticipated. In CTEPH patients at Amsterdam UMC, aPL testing was expected to be systematically implemented from 2017 onward; however, only a gradual implementation of standardized APS screening was observed ( **Supplementary Table S5** (online only)). 3 This could have resulted in selection bias. As a result, not all aims could be answered completely. Nonetheless, with the available data, we demonstrate an APS prevalence approximating the prevalence reported by Jiang et al. and Miard et al. Furthermore, the well-defined APS group allowed for preliminary insights into the clinical consequences of identifying APS in patients with CTEPH. These results highlight an awareness of APS testing among Dutch CTEPH clinicians. Finally, a substantial proportion of patients did not undergo confirmatory testing after an initial positive aPL result, which again may have introduced a degree of selection bias. Most possible APS patients had single aPL positivity. It remains unclear whether the decision not to perform confirmatory testing was influenced by these serological findings. Nevertheless, no major differences in factors such as age, recurrent VTE, or other autoimmune disorders were observed among patients without APS ( Table 1 ), patients with single aPL positivity, and those who were not tested ( **Supplementary Table S1** (online only)).

In this real-world cohort of CTEPH and CTEPD without PH patients, we found a modest APS prevalence of 6%, of whom a substantial part were triple positive. CTEPH patients with APS were relatively young, had recurrent VTE more often, and mild thrombocytopenia was frequently observed post-PEA. Both proximal and distal disease occurred in APS patients.

## Data Availability

Data will not be available during the study inclusion period. Following publication, the data will be made available upon reasonable request to the corresponding author.

## Supplementary Material

Ergänzendes Material Supplementary Material

## References

[1] Delcroix M Torbicki A Gopalan D ERS statement on chronic thromboembolic pulmonary hypertension Eur Respir J 2021 57 06 2.002828E6 10.1183/13993003.02828-2020 33334946

[2] Guth S Wiedenroth C B Rieth A Exercise right heart catheterisation before and after pulmonary endarterectomy in patients with chronic thromboembolic disease Eur Respir J 2018 52 03 1.800458E6 10.1183/13993003.00458-2018 30139773

[3] Humbert M Kovacs G Hoeper M M 2022 ESC/ERS guidelines for the diagnosis and treatment of pulmonary hypertension Eur Respir J 2023 61 01 2023 10.1183/13993003.00879-2022 36028254

[4] Fedullo P F Auger W R Kerr K M Rubin L J Chronic thromboembolic pulmonary hypertension N Engl J Med 2001 345 20 1465 1472 11794196 10.1056/NEJMra010902

[5] Pepke-Zaba J Delcroix M Lang I Chronic thromboembolic pulmonary hypertension (CTEPH): results from an international prospective registry Circulation 2011 124 18 1973 1981 21969018 10.1161/CIRCULATIONAHA.110.015008

[6] Ende-Verhaar Y M Meijboom L J Kroft L JM Usefulness of standard computed tomography pulmonary angiography performed for acute pulmonary embolism for identification of chronic thromboembolic pulmonary hypertension: results of the InShape III study J Heart Lung Transplant 2019 38 07 731 738 30962147 10.1016/j.healun.2019.03.003

[7] van Kan C van J Idrissi H E Clinical and radiologic characteristics of chronic thromboembolic pulmonary hypertension patients without a history of acute venous thromboembolism J Thromb Haemost 2025 23 06 1920 1926 40056989 10.1016/j.jtha.2025.02.033

[8] Klok F A Delcroix M Bogaard H J Chronic thromboembolic pulmonary hypertension from the perspective of patients with pulmonary embolism J Thromb Haemost 2018 16 06 1040 1051 29608809 10.1111/jth.14016

[9] Luijten D Talerico R Barco S Incidence of chronic thromboembolic pulmonary hypertension after acute pulmonary embolism: an updated systematic review and meta-analysis Eur Respir J 2023 62 01 2.300449E6 10.1183/13993003.00449-2023 37321620

[10] Manz X D Szulcek R Pan X Epigenetic modification of the von Willebrand factor promoter drives platelet aggregation on the pulmonary endothelium in chronic thromboembolic pulmonary hypertension Am J Respir Crit Care Med 2022 205 07 806 818 35081007 10.1164/rccm.202109-2075OC

[11] Delcroix M Kerr K Fedullo P Chronic thromboembolic pulmonary hypertension. Epidemiology and risk factors Ann Am Thorac Soc 2016 13(Suppl 3)S201 S206 27571001 10.1513/AnnalsATS.201509-621AS

[12] Barbhaiya M Zuily S Naden R The 2023 ACR/EULAR antiphospholipid syndrome classification criteria. Arthritis Rheumatology 2023 75 10 1687 1702 10.1002/art.42624 37635643

[13] Miyakis S Lockshin M D Atsumi T International consensus statement on an update of the classification criteria for definite antiphospholipid syndrome (APS) J Thromb Haemost 2006 4 02 295 306 16420554 10.1111/j.1538-7836.2006.01753.x

[14] Lang I Chronic thromboembolic pulmonary hypertension: a distinct disease entity Eur Respir Rev 2015 24 136 246 252 26028636 10.1183/16000617.00001115 PMC9487810

[15] Humbert M Kovacs G Hoeper M M 2022 ESC/ERS guidelines for the diagnosis and treatment of pulmonary hypertension Eur Heart J 2022 43 38 3618 3731 36017548 10.1093/eurheartj/ehac237

[16] Konstantinides S V Meyer G Becattini C 2019 ESC guidelines for the diagnosis and management of acute pulmonary embolism developed in collaboration with the European Respiratory Society (ERS) Eur Heart J 2020 41 04 543 603 31504429 10.1093/eurheartj/ehz405

[17] Simonneau G Azarian R Brenot F Dartevelle P G Musset D Duroux P Surgical management of unresolved pulmonary embolism. A personal series of 72 patients Chest 1995 107(1, Suppl)52S 55S 7813330 10.1378/chest.107.1_supplement.52s

[18] Auger W R Permpikul P Moser K M Lupus anticoagulant, heparin use, and thrombocytopenia in patients with chronic thromboembolic pulmonary hypertension: a preliminary report Am J Med 1995 99 04 392 396 7573095 10.1016/s0002-9343(99)80187-9

[19] Wolf M Boyer-Neumann C Parent F Thrombotic risk factors in pulmonary hypertension Eur Respir J 2000 15 02 395 399 10706510 10.1034/j.1399-3003.2000.15b28.x

[20] Wong C L Szydlo R Gibbs S Laffan M Hereditary and acquired thrombotic risk factors for chronic thromboembolic pulmonary hypertension Blood Coagul Fibrinolysis 2010 21 03 201 206 20182352 10.1097/MBC.0b013e328331e664

[21] D’Armini A M Totaro P Nicolardi S Impact of high titre of antiphospholipid antibodies on postoperative outcome following pulmonary endarterectomy Interact Cardiovasc Thorac Surg 2010 10 03 418 422 19934162 10.1510/icvts.2009.221630

[22] Park S Y Lee S M Shin J W Epidemiology of chronic thromboembolic pulmonary hypertension in Korea: results from the Korean registry Korean J Intern Med 2016 31 02 305 312 26689916 10.3904/kjim.2014.122 PMC4773708

[23] Colorio C C Martinuzzo M E Forastiero R R Pombo G Adamczuk Y Carreras L O Thrombophilic factors in chronic thromboembolic pulmonary hypertension Blood Coagul Fibrinolysis 2001 12 06 427 432 11555695 10.1097/00001721-200109000-00002

[24] Martinuzzo M E Pombo G Forastiero R R Cerrato G S Colorio C C Carreras L O Lupus anticoagulant, high levels of anticardiolipin, and anti-beta2-glycoprotein I antibodies are associated with chronic thromboembolic pulmonary hypertension J Rheumatol 1998 25 07 1313 1319 9676762

[25] Jiang X Du Y Cheng C Y Antiphospholipid syndrome in chronic thromboembolic pulmonary hypertension: a well-defined subgroup of patients Thromb Haemost 2019 119 09 1403 1408 31226720 10.1055/s-0039-1692428

[26] Cheng C Y Zhang Y X Denas G Du Y Jing Z C Pengo V Prevalence of antiphospholipid (aPL) antibodies among patients with chronic thromboembolic pulmonary hypertension: a systematic review and meta-analysis Intern Emerg Med 2019 14 04 521 527 30603858 10.1007/s11739-018-02021-z

[27] Heit J A Epidemiology of venous thromboembolism Nat Rev Cardiol 2015 12 08 464 474 26076949 10.1038/nrcardio.2015.83 PMC4624298

[28] Pang W Zhang Z Wang Z Higher incidence of chronic thromboembolic pulmonary hypertension after acute pulmonary embolism in Asians than in Europeans: a meta-analysis Front Med 2021 8 721294 10.3389/fmed.2021.721294 PMC8575791 34765615

[29] Mok C C Chan P T Ho L Y Yu K L To C H Prevalence of the antiphospholipid syndrome and its effect on survival in 679 Chinese patients with systemic lupus erythematosus: a cohort study Medicine 2013 92 04 217 222 23793109 10.1097/MD.0b013e31829cae47 PMC4553973

[30] Miard C Genty T Louvain-Quintard V Rezaiguia-Delclaux S Fadel E Stéphan F High antiphospholipid antibody titers and outcomes of pulmonary endarterectomy: a single-center retrospective observational cohort study J Heart Lung Transplant 2025 44 12 1996 2007 40907841 10.1016/j.healun.2025.08.017

[31] Alturaif N Alduraibi F K Marquez K Pulmonary endarterectomy in antiphospholipid syndrome: a retrospective analysis from the Saudi pulmonary hypertension registry Front Med 2026 13 1.736115E6 10.3389/fmed.2026.1736115 PMC12848920 41613608

[32] Erkan D Leibowitz E Berman J Lockshin M D Perioperative medical management of antiphospholipid syndrome: hospital for special surgery experience, review of literature, and recommendations J Rheumatol 2002 29 04 843 849 11950031

[33] Devreese K MJ de P G de B Guidance from the scientific and standardization committee for lupus anticoagulant/antiphospholipid antibodies of the international society on thrombosis and haemostasis: update of the guidelines for lupus anticoagulant detection and interpretation J Thromb Haemost 2020 18 11 2828 2839 33462974 10.1111/jth.15047

[34] Jamieson S W Kapelanski D P Sakakibara N Pulmonary endarterectomy: experience and lessons learned in 1,500 cases Ann Thorac Surg 2003 76 05 1457 1462discussion 1462–1464 14602267 10.1016/s0003-4975(03)00828-2

[35] Mayer E Jenkins D Lindner J Surgical management and outcome of patients with chronic thromboembolic pulmonary hypertension: results from an international prospective registry J Thorac Cardiovasc Surg 2011 141 03 702 710 21335128 10.1016/j.jtcvs.2010.11.024

[36] van Thor M CJ Lely R J Braams N J Safety and efficacy of balloon pulmonary angioplasty in chronic thromboembolic pulmonary hypertension in the Netherlands Neth Heart J 2020 28 02 81 88 31782109 10.1007/s12471-019-01352-6 PMC6977797

[37] Braams N J Kianzad A van J Long-term effects of pulmonary endarterectomy on right ventricular stiffness and fibrosis in chronic thromboembolic pulmonary hypertension Circ Heart Fail 2023 16 10 e010336 37675561 10.1161/CIRCHEARTFAILURE.122.010336 PMC10573098

[38] Kianzad A Baccelli A Braams N J Long-term effects of pulmonary endarterectomy on pulmonary hemodynamics, cardiac function, and exercise capacity in chronic thromboembolic pulmonary hypertension J Heart Lung Transplant 2024 43 04 580 593 38000764 10.1016/j.healun.2023.11.011

[39] Jenkins D Madani M Fadel E D’Armini A M Mayer E Pulmonary endarterectomy in the management of chronic thromboembolic pulmonary hypertension Eur Respir Rev 2017 26 143 160111 28298388 10.1183/16000617.0111-2016 PMC9489144

[40] Miranda S Park J Le G Prevalence of confirmed antiphospholipid syndrome in 18-50 years unselected patients with first unprovoked venous thromboembolism J Thromb Haemost 2020 18 04 926 930 31872492 10.1111/jth.14720

[41] Ordi-Ros J Sáez-Comet L Pérez-Conesa M Rivaroxaban versus vitamin K antagonist in antiphospholipid syndrome: a randomized noninferiority trial Ann Intern Med 2019 171 10 685 694 31610549 10.7326/M19-0291

[42] Cohen H Hunt B J Efthymiou M RAPS trial investigators. Rivaroxaban versus warfarin to treat patients with thrombotic antiphospholipid syndrome, with or without systemic lupus erythematosus (RAPS): a randomised, controlled, open-label, phase 2/3, non-inferiority trial Lancet Haematol 2016 3 09 e426 e436 27570089 10.1016/S2352-3026(16)30079-5 PMC5010562

[43] Pengo V Denas G Zoppellaro G Rivaroxaban vs warfarin in high-risk patients with antiphospholipid syndrome Blood 2018 132 13 1365 1371 30002145 10.1182/blood-2018-04-848333

[44] Puebla-Aldama D Cueto-Robledo G Barragan-Martinez M D Review of functional status and hemodynamic parameters in patients diagnosed with chronic thromboembolic pulmonary hypertension (CTEPH) with and without antiphospholipid syndrome (APLS) Curr Probl Cardiol 2023 48 07 101154 35192873 10.1016/j.cpcardiol.2022.101154

[45] Galli M Luciani D Bertolini G Barbui T Lupus anticoagulants are stronger risk factors for thrombosis than anticardiolipin antibodies in the antiphospholipid syndrome: a systematic review of the literature Blood 2003 101 05 1827 1832 12393574 10.1182/blood-2002-02-0441

[46] Taş S Antal A Durusoy A F Pulmonary endarterectomy in patients with antiphospholipid syndrome-associated chronic thromboembolic pulmonary hypertension Anatol J Cardiol 2022 26 05 394 400 35552176 10.5152/AnatolJCardiol.2021.1138 PMC9366407

[47] Camous J Decrombecque T Louvain-Quintard V Doubine S Dartevelle P Stéphan F Outcomes of patients with antiphospholipid syndrome after pulmonary endarterectomy Eur J Cardiothorac Surg 2014 46 01 116 120 24362260 10.1093/ejcts/ezt572

[48] Li C Zhao J Liu S Pulmonary thromboendarterectomy is a curative resolution for chronic thromboembolic pulmonary hypertension associated with antiphospholipid syndrome: a retrospective cohort study Lupus 2018 27 14 2206 2214 30451640 10.1177/0961203318810427

[49] Field C O Sarkar A Bdeir K Antiphospholipid syndrome (APS) is a platelet factor 4 (PF4)-centric immunothrombotic disorder bioRxiv 2025Preprint 10.1101/2025.11.07.687095 PMC13728500 42378231

[50] d’Amours L Bhattacharyya M Li Cai C Y Shekarsarai C Pepke-Zaba J Bunclark K Comments and opinions regarding “high antiphospholipid antibody titers and outcomes of pulmonary endarterectomy: a single-center retrospective observational cohort study” by Miard et al. J Heart Lung Transplant 2026 45 07 1165 1166 41823896 10.1016/j.healun.2026.02.1669

[51] Rosen K Raanani E Kogan A Chronic thromboembolic pulmonary hypertension in patients with antiphospholipid syndrome: risk factors and management J Heart Lung Transplant 2022 41 02 208 216 34836752 10.1016/j.healun.2021.10.016

[52] Ambati A Zuo Y Knight J S An update on inflammation in antiphospholipid syndrome Curr Opin Rheumatol 2023 35 02 89 97 36580355 10.1097/BOR.0000000000000926 PMC9877197

[53] Patriarcheas V Tsamos G Vasdeki D Antiphospholipid syndrome: a comprehensive clinical review J Clin Med 2025 14 03 733 39941405 10.3390/jcm14030733 PMC11818257

[54] Baranga L Khanuja S Scott J A In situ pulmonary arterial thrombosis: literature review and clinical significance of a distinct entity AJR Am J Roentgenol 2023 221 01 57 68 36856299 10.2214/AJR.23.28996

[55] Giannakopoulos B Krilis S A The pathogenesis of the antiphospholipid syndrome N Engl J Med 2013 368 11 1033 1044 23484830 10.1056/NEJMra1112830

[56] Chen M Wu Q Shao N The significance of CD16+ monocytes in the occurrence and development of chronic thromboembolic pulmonary hypertension: insights from single-cell RNA sequencing Front Immunol 2024 15 1.44671E6 10.3389/fimmu.2024.1446710 PMC11347785 39192976

[57] Sharma S Hofbauer T M Ondracek A S Neutrophil extracellular traps promote fibrous vascular occlusions in chronic thrombosis Blood 2021 137 08 1104 1116 33512471 10.1182/blood.2020005861

[58] Zuily S Domingues V Suty-Selton C Antiphospholipid antibodies can identify lupus patients at risk of pulmonary hypertension: a systematic review and meta-analysis Autoimmun Rev 2017 16 06 576 586 28411166 10.1016/j.autrev.2017.04.003

