# Supplementary material for: Antiphospholipid Syndrome in Chronic Thromboembolic Pulmonary Hypertension and Chronic Thromboembolic Pulmonary Disease without Pulmonary Hypertension: Prevalence and Clinical Impact in a Dutch Cohort
Source: TH Open. 2026 Sep 4;10:a29386324. doi: 10.1055/a-2938-6324 (PMC13551461; doi:10.1055/a-2938-6324)
Supplement: Supplementary file 1 [file 10-1055-a-2938-6324_29502744.pdf]

SUPPLEMENTS

	Single positive aPL (N = 18)	Not tested (N = 119)
**Age at diagnosis** (median, range)	71 (24-80)	69 (17-94)
**Female**	6 (33)	55 (46)
**BMI** (median, IQR)	26 (25-18)	27 (24-30)
**Anticoagulation**		
DOAC	15 (83)	94 (79)
VKA	3 (17)	25 (21)
**Smoker**		
Never	8 (44)	54 (45)
Current + Former	10 (56)	61 (51)
Unknown	0	4 (3)
**Blood group, non-O**	9 (50)	33 (28)
**Malignancy**	1 (6)	16 (13)
**Pulmonary comorbidities**[a]	6 (33)	35 (29)
**Other auto-immune disorder**	2 (11)	11 (9)
**Diabetes**	2 (11)	15 (13)
**Splenectomy**	0	0
**Thyroid hormone suppletion**	3 (17)	11 (9)
**Systemic hypertension**	8 (44)	46 (39)
**Cardiac comorbidities**		
Atrial fibrillation	2 (11)	6 (5)
Left-sided heart failure	0	2 (2)
Ischemic heart disease	1 (6)	3 (3)
Other	1 (6)	12 (10)
**TIA or iCVA**	0	9 (8)
**History of reported acute VTE**		
PE	14 (78)	90 (76)
DVT	5 (28)	28 (24)
Recurrent VTE	7 (39)	36 (30)
No history VTE	3 (17)	24 (20)

Supplementary Table S1. Baseline characteristics for patients with positive aPL once without repeated confirmation, and patients who were not tested for aPL

Data are presented as N (%), unless stated otherwise

[a] Chronic obstructive pulmonary disease (COPD), asthma, obstructive sleep apnea (OSA), interstitial lung disease

aPL, antiphospholipid antibodies; APS, antiphospholipid syndrome; BMI, body mass index; DOAC, direct oral anticoagulant; DVT, deep venous thrombosis; iCVA, ischemic cerebrovascular accident; IQR, interquartile range; PE, pulmonary embolism; TIA, transient ischemic attack; VKA, vitamin K antagonist; VTE, venous thromboembolism

	**Single positive aPL** (N = 18)	**Not tested** (N = 119)
**WHO FC**[a]		
II	5 (27)	40 (34)
III	11 (61)	71 (60)
IV	2 (11)	6 (5)
**NT-proBNP** (ng/L)[b]	407 (136-1012)	521 (163-1963)
**RHC**		
mRAP (mmHg)[b]	8 (5-10)	7 (5-10)
mPAP (mmHg)[b]	43 (12)	42 (14)
PAWP (mmHg)[c]	11 (3)	12 (4)
CI (L/min/m²) [c]	3.0 (0.8)	2.7 (0.8)
PVR (dyn·s·cm⁻⁵)[b]	447 (272-610)	449 (255-773)
SaO2 (%)[c]	92 (3)	93 (4)
SvO2 (%)[c]	66 (8)	64 (10)
**CTEPD without PH**[a]	1 (6)	12 (10)

Supplementary Table S2. Hemodynamic and functional parameters at baseline for patients with positive aPL once without repeated confirmation, and patients who were not tested for aPL

[a] N (%)
[b] Median (interquartile range)
[c] Mean (standard deviation)

aPL, antiphospholipid antibodies; APS, antiphospholipid syndrome; CI, cardiac index; CTEPD, chronic thromboembolic pulmonary disease; mPAP, mean pulmonary artery pressure; mRAP, mean right atrial pressure; PAWP, pulmonary artery wedge pressure; PH, pulmonary hypertension; PVR, pulmonary vascular resistance; SaO2, arterial oxygen saturation; SvO2, venous oxygen saturation; WHO FC, World Health Organization Functional Class.

Total cohort		
	**APS** (N = 9)	**No APS** (N = 112)
**WHO FC**[a]		
II	1 (11)	32 (29)
III	8 (89)	76 (68)
IV	0	4 (4)
**NTproBNP** (ng/L)[b]	212 (142- 168)	553 (112-1691)
**RHC**		
mRAP (mmHg)[b]	6 (6-8)	7 (5-10)
mPAP (mmHg)[b]	41 (15)	43 (14)
PAWP (mmHg)[c]	11 (4)	11 (3)
CI (L/min/m²)[c]	2.8 (1.1)	2.6 (0.7)
PVR (dyn·s·cm^{-5})[b]	382 (204-676)	468 (259-757)
SaO2 (%)[c]	95 (4)	92 (4)
SvO2 (%)[c]	64 (12)	63 (9)
**CTEPD without PH**	2 (22)	11 (10)
**Only CTEPH patients**		
	**APS** (N = 7)	**No APS** (N = 101)
**WHO FC**[a]		
II	1 (14)	27 (27)
III	6 (86)	70 (69)
IV	0	4 (4)
**NTproBNP**[b] (ng/L)	212 (120-2368)	757 (158-1781)
**RHC**		
mRAP (mmHg)[b]	6 (6-9)	7 (5-10)
mPAP (mmHg)[b]	46 (14)	45 (13)
PAWP (mmHg)[c]	10 (5)	11 (3)
CI (L/min/m²)[c]	2.4 (1.0)	2.5 (0.6)
PVR (dyn·s·cm^{-5})[b]	515 (337-923)	491 (325-839)
SaO2 (%)[c]	93 (3)	92 (4)
SvO2 (%)[c]	59 (6)	62 (8)

Supplementary Table S3. Hemodynamic and functional parameters at baseline (diagnosis) for the total cohort and only for patients with CTEPH (patients with CTEPD without PH are excluded)

[a] N (%)
[b] Median (interquartile range)
[c] Mean (standard deviation)

APS, antiphospholipid syndrome; CI, cardiac index; CTEPD, chronic thromboembolic pulmonary disease; CTEPH, chronic thromboembolic pulmonary hypertension; mPAP, mean pulmonary artery pressure; mRAP, mean right atrial pressure; PAWP, pulmonary artery wedge pressure; PH, pulmonary hypertension; PVR, pulmonary vascular resistance; SaO2, arterial oxygen saturation; SvO2, venous oxygen saturation; WHO FC, World Health Organization Functional Class.

	**Possible APS** (N = 19)
**Profile**	
Single	16 (84%)
Double	2 (11%)
Triple	1 [a] (5%)
**Lupus anticoagulant (LAC)**	
Positive	7 (37%)
Negative	12 (63%)

Supplementary Table S4. Specification of possible antiphospholipid syndrome (APS) patients

Patients that tested positive for one or more antiphospholipid antibodies once, without repeated confirmation of positive antibody test (n=18) or with repeated positive antiphospholipid antibodies but without meeting APS classification criteria (n=1). Data are presented as N (%)

[a] This patient passed away before repeated antiphospholipid antibody testing could be performed

	**2017** N = 35	**2018** N = 40	**2019** N = 25	**2020** N = 40	**2021** N = 32	**2022** N = 32	**2023** N = 36	**2024** N = 20
**aPL tested**	10 (29%)	17 (43%)	13 (52%)	24 (60%)	20 (63%)	15 (47%)	25 (69%)	16 (80%)
**2x positive aPL**	1	1	0	2	1	1	3	1
**1x positive aPL**	1	4	2	2	4	2	2	1
**aPL negative**	8	12	11	20	15	12	20	14

Supplementary Table S5. Gradual implementation of antiphospholipid antibody (aPL) testing
